# Serendipitous identification of natural Intergenotypic recombinants of hepatitis C in Ireland

**DOI:** 10.1186/1743-422X-3-95

**Published:** 2006-11-15

**Authors:** Isabelle Moreau, Susan Hegarty, John Levis, Patrick Sheehy, Orla Crosbie, Elizabeth Kenny-Walsh, Liam J Fanning

**Affiliations:** 1 Molecular Virology Diagnostic & Research Laboratory, Department of Medicine, Clinical Sciences Building, Cork University Hospital, Cork, Ireland; 2 Department of Gastroenterology, Cork University Hospital, Cork, Ireland

## Abstract

**Background:**

Recombination between hepatitis C single stranded RNA viruses is a rare event. Natural viable intragenotypic and intergenotypic recombinants between 1b-1a, 1a-1c and 2k-1b, 2i-6p, respectively, have been reported. Diagnostically recombinants represent an intriguing challenge. Hepatitis C genotype is defined by interrogation of the sequence composition of the 5' untranslated region [5'UTR]. Occasionally, ambiguous specimens require further investigation of the genome, usually by interrogation of the NS5B region. The original purpose of this study was to confirm the existence of a suspected mixed genotype infection of genotypes 2 and 4 by clonal analysis at the NS5B region of the genome in two specimens from two separate individuals. This initial identification of genotype was based on analysis of the 5'UTR of the genome by reverse line probe hybridisation [RLPH].

**Results:**

The original diagnosis of a mixed genotype infection was not confirmed by clonal analysis of the NS5B region of the genome. The phylogenetic analysis indicated that both specimens were natural intergenotypic recombinant forms of HCV. The recombination was between genotypes 2k and 1b for both specimens. The recombination break point was identified as occurring within the NS2 region of the genome.

**Conclusion:**

The viral recombinants identified here resemble the recombinant form originally identified in Russia. The RLPH pattern observed in this study may be a signature indicative of this particular type of intergenotype recombinant of hepatitis C meriting clonal analysis of NS2.

## Background

Hepatitis C virus infects approximately 170 million individuals' world wide [1]. Chronicity develops in 50–80% of infections [2,3]. Hepatitis C exists as a family of viruses, divided into 6 genotypes each with multiple subtypes [4]. The management and treatment of chronic hepatitis C virus [HCV] is in part guided by the genotype of the infecting virus [5,6]. The requirement for liver biopsy can be guided by genotype. Current therapeutic options of pegylated interferon and ribavirin have a population efficacy of only 50% [7]. Genotype 2 and 3 respond with efficacy of 80% in clinical trials. Duration of therapy is determined by genotype. Individuals infected with either genotype 1 or 4 usually have to undergo treatment for 48 weeks, while genotypes 2 and 3 usually have treatment duration of 24 weeks. There are several methods which can be used to determine viral genotype among which, (a) DNA sequencing of amplicons from the 5'untranslated region [5'UTR] (b) restriction length fragment polymorphism analysis and (c) Reverse Line Probe Hybridization [RLPH] [8]. Determination of viral genotype using RLPH can readily identify rare mixed genotype infections [9]. On occasion non-specific reactivity between probes and amplicons can give an indication of an apparent mixed genotype infection. However, upon repeat with additional specimens these ambiguities usually resolve to a single genotype infection. RLPH may infrequently yield ambiguous patterns which required further investigation. It may be possible to discriminate between very similar isolates by extension of the analysis to additional regions of the genome [10]. Alternatively, there are genotype specific motifs present within NS5B which can then be used to delineate strain genotype.

## Results and Discussion

**Figure 1:**
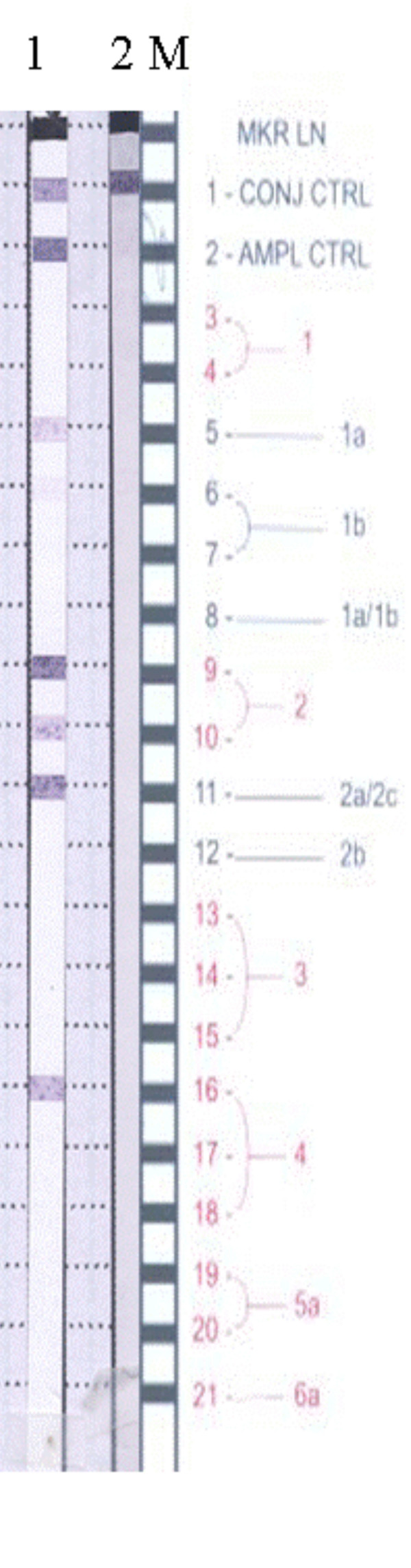
The 5'UTR amplicon used in the RLPH assay was generated using the Roche Diagnostics COBAS AMPLICOR HCV MONITOR kit. Hybridization and subsequent chromogenic development were performed as per AutoLipa-Versant protocol. Lane 1, HC9A98987; Lane 2, negative control; M, Interpretation chart; CONJ CTRL, conjugate control; AMP CTRL, PCR amplification control. The HCV specific banding pattern observed was 5, 6 (weak), 9–11, 16. A banding pattern of 5, 9–11 is indicative of a genotype 2a/2c infection, while banding at positions 6 and 16 are usually indicative of a genotype 4a infection and banding at positions 5 and 16 can be indicative of a genotype 4e. The nitrocellulose strip most proximal to the interpretation chart is the negative control.

Following routine RLPH of specimen HC9A98987 it was noticed that an unusual banding pattern indicative of a putative genotype 2 and 4 mixed infection was evident [Figure 1]. This interpretation is based on the fact that the banding at positions 9, 10, and 11 are indicative of a genotype 2a/c. However, a specimen of genotype 2a/c can also have a pattern of 5, 9, 10 and 11. In addition, a banding pattern of 6 and 16 is usually indicative of a genotype 4a, also, 5 and 16 is indicative of a specimen of genotype 4e. There is no banding pattern to support the existence of a genotype 1 within this isolate. The genotype determination was repeated on several occasions for this specimen and on an additional specimen from the same patient (results not shown) all of which yielded the same banding pattern. The DNA sequence analysis of the 5'UTR indicated that the specimen was of genotype 2, GenBank [DQ417427], with no definable subtype or indication of a mixed genotype infection with a strain of genotype 4. In light of this fact, clonal analysis of the NS5B region from HC9A98987 was undertaken. 14 clones were sequenced [DQ417439–DQ417452]. A search for similarities with previously published sequences was performed with the program Blast. [11] The results indicated that this region of the genome had homology to sequences of HCV 1b [e.g., D50485, AF31916] and not the expected genotype 2 or 4. The corresponding amino acid sequences analysis indicated that there were only 7 unique isolates, GenBank [DQ417439, DQ417443, DQ417446, DQ417447, DQ417449, DQ417451, DQ417452].

Subsequent clonal analysis of (i) the core sequences [DQ417428–DQ417433] and (ii) the HVR1 sequences [DQ417434–DQ417438] indicated that these regions of the genome were in fact homologous to viruses within the 2k subtype [e.g., AB031663]. Two clones from the core region had synonymous mutations [DQ417430, DQ417433]. The HCV 2k [e.g., AB031663] isolate has a relatively high prevalence in the countries of the former USSR. Kalinina *et al *reported in 2002 the identification of a natural intergenotypic recombinant form of HCV which had homology to 2k at the 5' end of the genome and to 1b at the 3' end of the genome [12]. The cross over point was identified as occurring within the NS2 region by Simplot by Kalinina [12].

**Figure 2:**
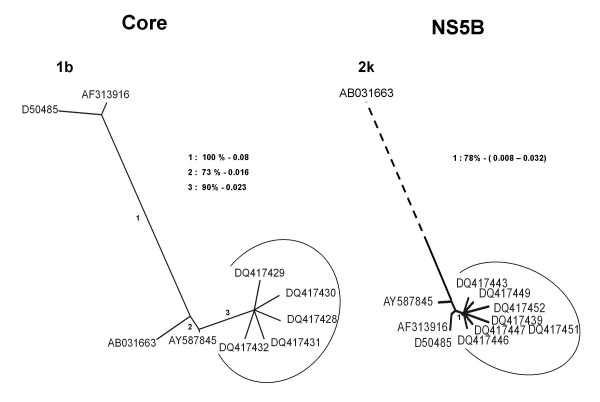
Phylogenetic tree generated by NEIGHBOR program in the PHYLIP package for NS5B and core amino acid sequences. Genetic distance was calculated with PRODIST program from the PHYLIP package based on Kimura's distance. A bootstrap analysis using 100 bootstrap replicates was performed to assess the reliability of each branch point. Bootstrap values greater than 70% are shown. The corresponding genetic distances are also shown. The arc signifies the sequences generated from HC9A98987. The dashed line reflects a genetic distance of 0.29 between AY587845 (2k/1b) and AB031663 (2k).

Following these observations, the core, the HVR1 and the NS5B amino acid sequences from HC9A98987 were aligned against reference strains of different genotypes [AY587845 (2k/1b), AB031663 (2k), AF313916 (1b) and D50485 (1b)] and a phylogenetic analysis was carried out. Phylogenetic trees were constructed with the NEIGHBOR program in the PHYLIP package and genetic distances were calculated with PRODIST program in the PHYLIP package based on Kimura's distance. [13] The results indicated clearly that sequences corresponding to specimen HC9A98987 are related to genotype 2k in the core and HVR1 region (data not shown) and closely related to genotype 1b in the NS5B region [Figure 2]. All of these three regions are highly homologous with AY587845 (2k/1b) the recombinant form initially identified in Russia [12].

**Figure 3:**
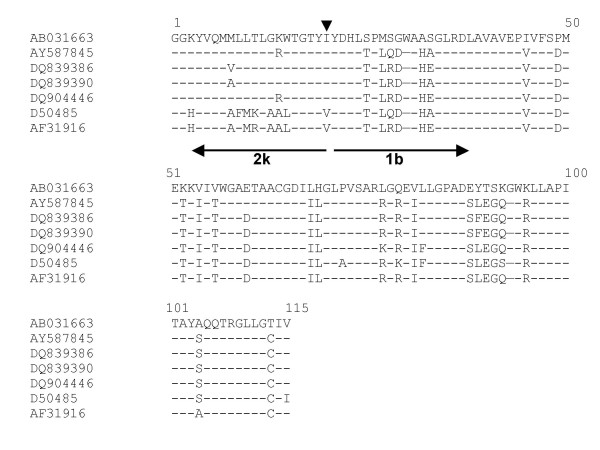
Amino acid alignment of sequences from sera (a) HC9A98987 [DQ839386, DQ839390] and (b) HC9A99966 [DQ904446], and reference strain [AB031663] (2k), [AY587845] (2k/1b), [D50485] (1b) and [AF313916] (1b) from the NS2 region. The recombination break point as identified by Kalinina is marked by the arrowhead. The directionality of the genotypes is indicated by the arrow, with subscripted genotype/subtype.

To investigate if specimen HC9A98987 was a possible recombinant form of HCV, primers were designed with the Primer3 program [14] to span the previously identified recombination point. A product of the expected 346 bp [3168–3413, reference strain [AY587845] (2k/1b)] was recovered by nested RT-PCR [Table 1]. The amplicon was cloned and the DNA sequence from 5 clones examined. Four of the 5 clones were found to be identical at the level of amino acid sequence [DQ839386–DQ839389] with a minor quasispecies [DQ839390]. Sequence analysis indicated that this region of specimen HC9A98987 is homologous with the reference strain [AY587845] [Figure 3]. The recombinant form identified here likely shares the same cross over point as the RF_2k/1b reported by Kalinina [12]. This is the first independent identification of this natural intergenotypic recombinant of HCV between 2k/1b.

**Table 1:** Primer details for the amplification of core and NS2

**Region**	**Polarity**	**Primer sequence**	
**Core**^a^			**Position**^b^
Core A1	Sense	5'-GAGTGCCCCGGGAGGTCTCGTAGA-3'	309–332
Core A2	Antisense	5'-RCCRCAYGTRANGGTRTCGATGAC-3'	705–728
Core B1	Antisense	5'-GCRAARCCRCAYGTRANGGTRTCG-3'	710–733
Core B2	Sense	5'-GGGAGGTCTCGTAGACCGTGCA-3'	318–339

**NS2**			**Position**^c^
NS2 KOS	Sense	5'-GCAAGCTCTGTTGAGGATAT-3'	3025–3044
NS2 KOAS	Antisense	5'-TGTGAGGCTAGTGACGATGC-3'	3405–3424
NS2 KIS	Sense	5'-GGTGGCAAGTACGTCCAGAT-3'	3068–3087
NS2 KIAS	Antisense	5'-TGACGATGCAGCCAAGTAAG-3'	3394–3413

^a ^Y = C or T; R = A or G; N = A, T, G or C.

^b ^Reference strain NC_004102 (1a)

^c ^Reference strain AY587845 (2k/1b)

Following the identification of HC9A98987 as a recombinant form of HCV similar to AY587845 (2k/1b), the laboratory investigated whether an additional specimen (hence, HC9A99966) from another individual with the same RLPH pattern was also a candidate natural intergenotypic recombinant form. Clonal analysis of the NS2 region was performed on this specimen from the second patient (HC9A99966). The sequences of six clones were determined. All six clones were of identical nucleotide sequence [DQ904446] and were found by sequence analysis to be homologous to the natural intergenotypic recombinant related to HC9A98987, i.e., AY587845 (2k/1b) [Figure 3].

While, the prevalence of the 2k/1b recombinant form of HCV is globally low, its existence poses an interesting challenge as to what anti-viral regimen should be followed. Mechanistically the identification of a recombinant strain of HCV, a single stranded RNA virus, poses some interesting questions. The initial recombination event had to happen in a hepatocyte infected with a 2k and a 1b isolate. Recombination had to maintain the reading frame of the polyprotein. The majority of recombination events would be expected to yield fatal nucleotide lesions generating life cycle incompetent virions. It is interesting to hypothesize that perhaps the generation of this recombinant form of HCV is the result of "strand switching" during replication rather than classical recombination-ligation of viral RNA as previously proposed [15].

An additional natural intergenotypic recombinant between genotypes 2i and 6p has very recently been described [16]. The recombination break point was again narrowed down to a region at the 3' end of the NS2 gene, possibly extending into the NS3 gene. It is currently evident that the viable recombination occurs infrequently in HCV biology. The frequency of this phenomenon may be under estimated. In an attempt to estimate the prevalence of HCV recombinants Cristina and Colina recently reported the identification of an intragenotypic recombinant between 1a and 1c by examining only 89 full length sequences, representative of all genotypes present in the LANL database [17,18]. These results may give an insight into the actual prevalence of HCV recombinant forms but would require prospective examination to verify the findings. Molecular characterisation of these natural chimeras may yet yield some unique insights into life cycle of hepatitis C. Recombinant forms for HIV do not have the same sensitivity to anti-retroviral therapy as wild type HIV-1 clade B isolates [19]. The sensitivity of recombinant forms of HCV to pegylated interferon based therapy is unknown. Intergenotypic recombinant isolates that have genotype 1 as part of the genomic backbone will likely require a conservative approach, with patients being treated for 48 rather than 24 weeks.

## Conclusion

The diagnostic standard for determining HCV genotype, irrespective of methodology, is to interrogate the sequence of the 5'UTR. The serendipitous identification of two recombinant forms of HCV, related to the Russian 2k/1b recombinant form, in Ireland was the result of a detailed molecular investigation across the genome. The RLPH pattern observed in this study may be a signature indicative of this particular type of intergenotype recombinant of hepatitis C meriting clonal analysis of NS2. Perhaps diagnostic laboratories need to consider confirmation of genotype by interrogation of the NS5B to ensure that management of chronic HCV is optimal.

## Methods

### HCV Genotyping

The specimens referred to in this manuscript were received as part of the routine diagnostic services provided by the Molecular Virology Diagnostic and Research Laboratory to Cork University Hospital. The viral status of all specimens received by the diagnostic laboratory are qualitatively and quantitatively evaluated. Subsequent to confirmation of viraemia, specimens are routinely genotyped by RLPH (Versant-Bayer, UK) [20]. The specimens used during the study outlined here are HC9A98987 and HC9A99966, each from separate unrelated individuals originally from Russia and Georgia, respectively, but resident in Ireland at the time of sample acquisition.

### RT-PCR

The RNA (QIAGEN, UK. Qiamp viral RNA) was randomly primed to generate cDNA with AMV reverse transcriptase (Promega, USA). Taq DNA polymerase (Promega, USA) was used to amplify the core, HVR1 and NS5B amplicons. Pwo DNA polymerase (Roche, UK) was used to amplify the NS2 amplicon. The primers used to amplify the core and the NS2 regions are listed in Table 1. The primers used to amplify the HVR1 region were described by Farci *et al *[21]. The core, the HVR1 and the NS2 primary and secondary PCRs were carried out under the following conditions: 94°C for 3 min., 35 cycles of 94°C for 15 s, 60°C (core), 62°C (HVR1) 55°C (primary PCR NS2) or 58°C (secondary PCR NS2) for 30 s and elongation at 72°C for 45 s; a final elongation at 72°C for 10 min. completed the PCR. To amplify the NS5B region, a hemi-nested PCR was performed using primers Pr3, Pr4 first described by Morice *et al *and Pr5 designed by Sandres-Saune *et al *under the following PCR conditions: 95°C for 5 min., 35 cycles of 95°C for 30 s, 58°C (primary PCR with Pr3 and Pr4) or 55°C (secondary PCR with Pr3 and Pr5) for 30 s and elongation at 72°C for 30 s; a final elongation at 72°C for 10 min. completed the PCR [22,23]. 5'UTR sequence was determined following amplification of the region from position -299 to 6nt (based on prototype strain M632321).

### Clonal Analysis

The Taq DNA polymerase generated amplicons were cloned into DH5α by TA cloning (Invitrogen Belgium). The NS2 amplicon was cloned by use of the Zero Blunt TOPO system (Invitrogen, Belgium). DNA sequencing was out sourced to MWG, Germany.

Sequence similarities between the sequences generated during this study were examined by use of the BLASTN web program [11]. Sequence alignment was performed with CLUSTALW (version 1.74) [24]. Phylogenetic analyses were conducted by using the NEIGHBOR program in the PHYLIP package [13]. A boostrap analysis using 100 bootstrap replicates was tested. Genetic distances were calculated with PRODIST from the PHYLIP package based on Kimura's distance.

Reference strains used in the present study were all obtained from GenBank [AY587845 (genotype 2k/1b), AB031663 (genotype 2k), D50485 (genotype 1b) and AF31916 (genotype 1b)]. Nucleotide sequences reported in this study have been assigned the following accession numbers which were derived from HC9A98987 Genbank: [DQ417427] (5'UTR). [DQ417428 to DQ417433] (Core sequences), [DQ417434 to DQ417438] (HVR1 sequences), [DQ417439 to DQ417452] (NS5B sequences), [DQ839386 to DQ839390] (NS2 sequences). [DQ904446] was derived from the NS2 sequence from sera HC9A99966.

## List of abbreviations

Hepatitis C Virus HCV

Untranslated Region UTR

Reverse Line Probe Hybridisation RLPH

Human Immunodeficiency Virus HIV

## Competing interests

The author(s) declare that they have no competing interests.

## Authors' contributions

IM Carried out NS2 clonal analysis, sequence alignment and phylogenetic analysis. Preparation of manuscript

SH Performed core, HVR1 and NS5B sequence/cloning

JL Determined qualitative, quantitative and initial genotype of specimens described here

PS Assisted SH in RT-PCR and amplicons cloning

OC & EKW Clinicians who manage HCV at Cork University Hospital

LJF Supervised project and assisted with analysis and preparation of manuscript.
